# Personal social capital and self-rated health among middle-aged and older adults: a cross-sectional study exploring the roles of leisure-time physical activity and socioeconomic status

**DOI:** 10.1186/s12889-020-10043-6

**Published:** 2021-01-06

**Authors:** Youngdeok Kim, Tim Schneider, Eric Faß, Marc Lochbaum

**Affiliations:** 1 Department of Kinesiology and Health Sciences, Virginia Commonwealth University, Richmond, VA USA; 2 Faculty of Sport Science, Ruhr University Bochum, Bochum, Germany; 3 Department of Kinesiology and Sport Management, Texas Tech University, Lubbock, TX USA; 4 Education Academy, Vytautas Magnas University, Kaunas, Lithuania

**Keywords:** Bonding and bridging social capital, Perceived health, Aging

## Abstract

**Background:**

Personal social capital, which refers to the scope and quality of an individual’s social networks within a community, has received increasing attention as a potential sociological factor associated with better individual health; yet, the mechanism relating social capital to health is still not fully understood. This study examined the associations between social capital and self-rated health while exploring the roles of leisure-time physical activity (LTPA) and socioeconomic status (SES) among middle-aged and older adults.

**Methods:**

Cross-sectional data were collected from 662 middle-aged and older adults (Mean age: 58.11 ± 10.59 years old) using the Qualtrics survey panel. Personal Social Capital Scale was used to measure bonding and bridging social capital and the International Physical Activity Questionnaire was used to assess LTPA levels. SES was assessed by education and household income levels. Self-rated health was assessed using a single item, by which the participants were categorized into the two groups, having ‘good’ vs. ‘not good’ self-rated health. A series of univariate and multivariate logistic regression models were established to examine the independent and adjusted associations of social capital with self-rated health and to test mediating and moderating roles of LTPA and SES, respectively.

**Results:**

Bonding and bridging social capital were positively associated with self-rated health (Odds ratios = 1.11 and 1.09; *P*’s < .05, respectively), independent of LTPA that was also significantly associated with greater self-rated health (*P*-for-linear trends = .007). After adjusting SES, the associations of social capital were significantly attenuated and there was a significant interaction effect by household income (*P*-for-interaction = .012). Follow-up analyses stratified by household income showed that beneficial associations of social capital with self-rated health were more apparent among the people with low and high levels of household income; yet, LTPA was the stronger predictor of self-rated health among those in the middle class of household income.

**Conclusions:**

Findings suggest that both social capital and LTPA are associated with better self-rated health; yet, these associations vary by SES. The health policymakers should address both social capital and LTPA for enhancing perceived health among aging populations but may need to consider varying SES backgrounds.

**Supplementary Information:**

The online version contains supplementary material available at 10.1186/s12889-020-10043-6.

## Background

Aging is associated with numerous physical and biological changes, including a decrease in physiological reserves and a decline in physical, cognitive, and functional capacities, posing older adults at greater risk of experiencing health-related strains [1]. As a result, healthy aging becomes increasingly recognized as a key area of action within health policy worldwide to improve the quality of life among aging populations [2]. For the older adults, self-rated health, which refers to a self-assessment of an individual’s general health status [3], has been largely examined as one of the most relevant indicators of healthy aging [4]. Specifically, previous studies have reported self-rated health status to be a valid and sensitive predictor of mortality, physical performance or long-term care [5, 6], indicating self-rated health as one of the critical components of well-being in everyday life among the aging population [7, 8].

Given the critical role of self-rated health in healthy aging, there have been continuous efforts to explore its determinants at individual and socio-ecological levels. Social capital is one of the sociological factors that has received increasing attention in the past few decades as a potential determinant of health and well-being among elderly people [9]. Although there is a lack of consensus regarding its definition across the disciplines, the term social capital generally refers to social relations or connections and the productive benefits that arise from them [10]. Within the contexts of health, social capital has been conceptualized and studied at both individual and societal levels. The first approach emphasizes social capital as an individual attribute highlighting the role of a social network; whereas the second approach views social capital as a collective feature characterizing the entire social community. It has been hypothesized that individual and collective social capitals share a commonality in terms of the sources (e.g., network characteristics, macro-political structure) [11], but there has been an ongoing debate over the role of collective social capital as a determinant of health. Recently, a growing number of studies has demonstrated individual social capital as a stronger predictor of health in elderly people [12]; yet, the underlying mechanisms are not clearly understood.

One of the most prominent hypotheses explaining the links between individual-level social capital and health is the consideration of social influence on behaviors [13], such as the spread of information and norms regarding health-related behaviors [14]. Supporting an indirect pathway through the fostering of healthy behavior, PA has frequently been considered as a possible factor mediating the relationship between social capital and health [15]. Particularly, leisure-time PA (LTPA) is one of the modifiable lifestyle behaviors positively associated with the maintenance of health and well-being in elderly people [16]. LTPA is also a complex behavior influenced by multiple socio-ecological factors, in which social capital has been frequently examined as a significant predictor of PA [17, 18]. However, there is a little study that directly examined the role of LTPA on the relationship between individual-level social capital and self-rated health among older adults, requiring further investigation of a potential mechanism linking social capital and health in this population group.

Meanwhile, socioeconomic status (SES) is another important individual characteristic that has long been examined regarding the health of individuals. SES is a comprehensive measure of a person’s combined economic and social status and has been frequently operationalized along the dimensions of education, income, and occupation. For the older adults, there exists considerable evidence for an effect of SES on self-rated health, in which lower SES is associated with lower self-rated health [19], due to socioeconomic disadvantages and inequalities that may matter for the health. Such disadvantages include a lack of accessible resources to promote health-enhancing behaviors, resulting in SES being one of the key determinants of PA where the people with higher SES are more physically active than those with lower SES [20]. Additionally, it is shown that socioeconomically disadvantaged people tend to cluster with people who are in similar SES positions [21], indicating that social networks or support of certain socioeconomic groups may be more limited or resourceful than others and thus, social capital may be differently effects for persons with different SES. In line with this, there have been few studies exploring the interrelationship of socioeconomic inequalities in health as well as LTPA with social capital [22, 23]; yet, the evidence is inconclusive requiring further study to elucidate the role of SES on health-enhancing effects of social capital among aging populations.

Collectively, the current study aims to close the gaps identified in the literature by addressing the following purposes: a) to examine the independent association of personal social capital with self-rated health in middle-aged and older adults; b) to test the mediating role of LTPA on the relationship between social capital and self-rated health; and c) to extend analysis testing the moderating effects by SES characteristics.

## Methods

### Survey sample

A cross-sectional survey was conducted using a Qualtrics online survey platform (Qualtrics Inc., Provo, UT, USA). Qualtrics is a well-known online survey company that offers various services for the research, which includes access to the Qualtrics research panel facilitating participant recruitment for online data collection. The Qualtrics research panel consists of pre-arranged individuals recruited from various sources who have agreed to respond to Qualtrics online surveys in exchange for compensation. The Qualtrics research panel is increasingly recognized as an acceptable online data source for the research [24] and has been successfully used for health-related studies involving older adults [25]. For the present study, the recruitment of participants as well as the administration of the survey were operated by Qualtrics, Inc. Adults in the pool of Qualtrics research panel who met the following eligibility criteria were invited to the study: 1) adults aged ≥40 years old; 2) residents of Virginia in the U.S.; and 3) can read English. Upon electronic informed consent, participants were asked to complete the online survey including a set of questionnaires about demographic characteristics, social capital, and PA levels at their own pace and at any time or place where they can have internet access to complete the survey in either web- or mobile-based platforms. Of the initial respondents of 803, 17.56% (*n* = 141) were excluded due to the failure to pass the pre-designed quality check questions and to provide valid data on key variables, leaving the final sample of 662 (*Mean* age: 58.11 ± 10.59 years old; *Range* = 40–85 years old);. Participants who completed the survey received the compensation in various forms (e.g., gift cards, electronic coupons) from the Qualtrics Inc. The study protocol was approved by the Institutional Review Board at Texas Tech University (IRB#: 2019–307).

### Measures

#### Personal social capital

Individual-level social capital was assessed using the Personal Social Capital Scale (PSCS). PSCS was developed by Chen et al. [26] to measure the two subtypes of individual-level social capital, bonding and bridging social capital, that are derived from a network perspective. Bonding social capital refers to links between community residents whose social identities are similar while bridging social capital involves connections between community residents with differing status and power [11]. Thus, bonding social capital indicates the cohesion within a group, enhancing the homogeneity of the group’s characteristics and helping to mobilize reciprocity and solidarity among actors. By this means it strengthens the linkages between the members of the group. In contrast, bridging social capital is directed outside the group by linking actors of different networks. PSCS consists of 10 composite items calculated based on 42 subitems measured by a five-point Likert scale with 1 = ‘none or a few’ to 5 = ‘all or a lot’.

In the initial development, the scale’s internal consistency was proven to meet common standards with Cronbach’s alphas of 0.87 for the overall scale and 0.85 and 0.84 for the bonding and bridging social capital subscales, respectively, among 128 Chinese adults aged between 18 and 50 years old [26]. Additionally, the authors demonstrated acceptable construct validity by examining known-group differences as well as predictive validity. PSCS has repeatedly been tested for its validity and reliability across different population groups [27, 28] including older adults [29]. In the present study, the scale was checked for reliability with satisfying levels of internal consistency (Cronbach’s α = 0.82 & 0.81 for bonding and bridging scale, respectively). Additionally, confirmatory factor analysis was conducted and showed acceptable fit indices (Standardized root mean squared residual = 0.059; Root mean square error of approximation = 0.098; Comparative fit index = 0.911), indicating that the PSCS is an adequate tool to measure bonding and bridging social capital among the sample of this study.

#### Self-rated health

The outcome variable of self-rated health was measured with a single question, asking how they would rate their health in general using a five-point Likert scale ranged from 1 (poor) through 5 (excellent). Self-rated health question is a simple and easy to administer measure of general health, allowing respondents to prioritize and evaluate different aspects of their health. The self-rated health question has been widely used as a measure of self-rated health in the elderly population [30]. Although the reliability of the item has been debated in some contexts, its adequate predictive validity in terms of several objective health measures has been verified in numerous studies [31, 32]. For instance, Baćak and Ólafsdóttir [33] examined the concurrent validity of self-rated health item among 19 European countries and found the measure to be a significant predictor of both mental and physical health (0.386 < *r* < 0.768). For the present study, participants were categorized into ‘not-good’ (fair/poor) and ‘good’ (good/very good/excellent) health groups, which is in line with the majority of literature examined self-rated health with social capital [34].

#### Leisure-time physical activity

The long form of the International Physical Activity Questionnaire (IPAQ) was used to assess the level of PA. Initially developed by the International Consensus Group for the development of an internationally standardized measure of PA [35], the IPAQ has been extensively used worldwide with well-established evidence of the reliability and validity across different settings [36, 37]. The participants were asked to disclose frequency and time they have spent in PA at moderate- and vigorous-intensity levels during the past 7 days across the four domains (i.e., domestic, leisure-time, work-, and transport-related activities). For the present study, participant’s responses to leisure-time PA were used to calculate total energy expenditures in metabolic-equivalent units (METs-minutes/week) by the IPAQ scoring guidelines [35]. Participants were categorized into three groups based on the current recommendations [38]: ‘no LTPA’, ‘< 600 MET-minutes/week’, which is equivalent to 150 min of moderate PA, and ‘≥600 MET-minutes per week’.

#### Socioeconomic status

Following common operational definitions [39], SES was assessed among the dimensions of education and household income. Education level was assessed through a seven-point scale with the response categories of ‘less than high school diploma’, ‘high school degree’, ‘some college credit, no degree’, associate degree’, ‘bachelor’s degree’, ‘master’s degree’ and ‘doctorate’. Annual household income was assessed through a six-point scale with the response categories of ‘<$20 k’, ‘$20k to <$35k’, ‘$35k to <$50k’, ‘$50k to <$75k’, ‘$75k to <$100k’ and ‘≥$100 k’. Both variables were recoded to ‘low’ (‘high school degree’ or below), ‘middle’ (‘some college credit, no degree’ and ‘associate degree’) and ‘high’ (‘bachelor’s degree’ or above) for education level, and ‘low’ (<$35 k), ‘middle’ ($35 k to <$75 k) and ‘high’ (≥$75 k) for household income, respectively.

#### Other study covariates

Demographic characteristics including age (< 60 years old vs. ≥60 years old), gender (male vs. female), marital status (married or domestic partnership vs. single or no partnership), and ethnicity (white vs. others) were collected. Additionally, self-reported history of chronic diseases they have ever confirmed by health professionals such as stroke, asthma, cancer, arthritis, diabetes, kidney disease were used to categorize the participants into three groups (‘no’, ‘one’, and ‘one or more’ chronic diseases).

### Statistical analysis

Descriptive statistics of study variables were calculated differentiated by self-rated health status, followed by chi-square test of independence examining between-group differences in the proportions for categorical variables. The differences in social capital scores by the key explanatory variables including LTPA levels and SES characteristics were examined using one-way analysis of variance with Tukey’s pairwise comparisons. Additionally, given the ordered nature of LTPA and SES variables, linear trends were tested using orthogonal polynomial contrasts.

Using the self-rated health (‘good’ vs. ‘not good’) as a dependent variable, logistic regression model was established predicting the likelihoods of reporting ‘good’ self-rated health based on a set of explanatory variables. The models were hierarchically developed to address the proposed research questions. The model 1 was established to examine the association of social capital with self-rated health without adjustment of other study covariates, followed by the model 2 adjusting for the demographic variables including age, gender, ethnicity and marital status, as well as for the history of chronic diseases. LTPA levels were then introduced in the model 3 to test the possible mediating effect on the relationship between social capital and self-rated health as suggested by Baron and Kenny [40, 41]. Specifically, two nested logistic regression models (model 2 and 3) were statistically compared using the Karlson-Holm-Breen (KHB) method [42] to test the significance of the indirect effect (i.e., mediating effect of LTPA). Lastly, SES variables including education and household income were additionally included in the model 4. After applying the overall model, the possible moderating effects of SES were tested by including interaction terms between social capital and SES variables (i.e., bonding-by-education, bonding-by-household income, bridging-by-education, and bridging-by-household income) in the model. If the interaction effect is significant regarding the specific SES variable, follow-up simple effect logistic regression analyses were conducted to examine the differences in the effect structures by the level of SES variable. Throughout the analyses, the results are presented as odds ratio (OR) along with 95% confidence interval (CI). As a supplement, average marginal effects (AME) expressing the average influence of independent variable in a form of probability of reporting “Good” self-rated health was reported. Additionally, linear trends analyses were performed using orthogonal polynomial contrasts for the ordered categorical variables including LTPA and SES variables. The goodness of fit of the logistic models were reported with the Nagelkerke’s pseudo-*R*^2^, representing the reduction of deviance due to the inclusion of predictors, and the Hosmer-Lemeshow goodness-of-fit test. Multicollinearity was tested by the variance inflation factors for each model. All data analyses were performed using the SAS v9.4 (SAS Institute, Cary, NC, USA) and STATA v13.1 (Stata Corp, College Station, TX, USA). Statistical significance was set at *P* ≤ .05.

## Results

The final sample characteristics stratified by self-rated health are presented in Table 1. Overall, 76.4% (*n* = 506) participants were categorized into ‘good’ self-rated health group. A large percentage of sample was female (*n* = 507; 76.6%) or white ethnicity (*n* = 563; 85.0%). The chi-square tests of independence indicated significant differences of self-rated health by LTPA levels as well as SES characteristics (*P*’s < .001), in which the groups with greater LTPA levels or greater SES tended to have a relatively larger percentage of individuals reporting ‘good’ self-rated health. There were significant differences in both bonding and bridging social capital by LTPA levels and SES characteristics (Table 2), with significant linear trends in which greater LTPA and SES levels were associated with greater bonding and bridging social capital (*P*-for-linear trends <.001).

**Table 1:** Descriptive characteristics of study sample across self-rated health

Characteristics	Total	Self-rated health		*P*-value ^a^
		Good	Not good	
N (%)	662	506 (76.4)	156 (23.6)	
Gender (n, %)				.282
Male	155 (23.4)	113 (72.9)	42 (27.1)	
Female	507 (76.6)	393 (77.5)	114 (22.5)	
Age (n, %)				.070
< 60 years	363 (54.8)	267 (73.6)	96 (26.4)	
≥ 60 years	299 (45.2)	239 (79.9)	60 (20.1)	
Race/ethnicity (n, %)				.764
Non-Hispanic White	563 (85.0)	432 (76.7)	131 (23.3)	
Others	99 (15.0)	74 (74.7)	25 (25.3)	
Marital status (n, %)				.278
Married or partnership	375 (56.7)	293 (78.1)	82 (21.9)	
Single or no partnership	287 (43.4)	213 (74.2)	74 (25.8)	
Chronic diseases (n, %) ^b^				<.001
No	298 (45.1)	264 (88.6)	34 (11.4)	
Yes, one	226 (34.1)	173 (76.5)	53 (23.5)	
Yes, one or more	138 (20.8)	69 (50.0)	69 (50.0)	
LTPA (n, %)				<.001
No-LTPA	244 (36.8)	171 (70.1)	73 (29.9)	
< 600 MET-mins/wk	158 (23.8)	114 (72.2)	44 (27.8)	
≥ 600 MET-mins/wk	260 (39.4)	221 (85.0)	39 (15.0)	
Education (n, %) ^c^				<.001
Low	134 (20.3)	95 (70.9)	39 (29.1)	
Middle	257 (38.8)	185 (72.0)	72 (28.0)	
High	271 (40.9)	226 (83.4)	45 (16.6)	
Household income (n, %) ^d^				<.001
Low	200 (30.2)	131 (65.5)	69 (34.5)	
Middle	252 (38.1)	191 (75.8)	61 (24.2)	
High	210 (31.7)	184 (87.6)	26 (12.4)	

*LTPA* Leisure-time physical activity, *MET* Metabolic equivalent task

^a^*P*-value from the *x*^2^ test of independence

^b^Categorization was based on the response to the history of chronic diseases they have ever confirmed by health professionals including stroke, asthma, cancer, arthritis, diabetes, and kidney diseases

^c^low (‘high school degree’ or below), middle (‘some college credit, no degree’ and ‘associate degree’), and high (‘bachelor’s degree’ or above)

^d^low (<35*k*), *middle* (35k to <75*k*), *andhigh* (≥75k)

**Table 2:** Personal social capital scores by LTPA and SES characteristics

	Personal social capital	
	Bonding	Bridging
Total (*N* = 662)	14.08 (2.91)	11.38 (3.34)
LTPA		
No-LTPA	13.31 (3.02) ^a^	10.56 (3.35) ^a^
< 600 MET-mins/wk	14.27 (2.84)	11.95 (3.05)
≥ 600 MET-mins/wk	14.67 (2.68)	11.81 (3.35)
*P*-for-linear trend	<.001	<.001
Education		
Low	13.08 (2.83) ^c^	9.96 (3.13) ^b, c^
Middle	13.66 (2.93) ^c^	10.93 (3.16) ^c^
High	14.97 (2.67)	12.52 (3.23)
*P*-for-linear trend	<.001	<.001
Household income		
Low	12.93 (3.08) ^b, c^	10.50 (3.29) ^b, c^
Middle	14.05 (2.68) ^c^	11.41 (3.32) ^c^
High	15.21 (2.55)	12.19 (3.20)
*P*-for-linear trend	<.001	<.001

Values are presented as *Mean* (*Standard Deviation*) unless otherwise specified. All pairwise comparisons were adjusted by the Tukey’s method

*LTPA* Leisure-time physical activity, *MET* Metabolic equivalent tasks

^a^Significantly lower than ‘< 600 MET-mins/wk’ and ‘≥600 MET-mins/wk’ groups (*P* < .05)

^b^Significantly lower than ‘Middle’ group (*P* < .05)

^c^Significantly lower than ‘High’ group (*P* < .05)

The results of logistic regression analyses sequentially adjusting for study variables are presented in Table 3. Bonding social capital was positively associated with self-rated health (OR = 1.17; 95% CI = 1.08–1.26; AME = 0.026) in the unadjusted model 1. After adjusting for demographic variables as well as history of chronic diseases in model 2, both bonding (OR = 1.14; 95% CI = 1.04–1.24; AME = 0.019) and bridging (OR = 1.09; 95% CI = 1.01–1.18; AME = 0.013) social capital were positively associated with self-rated health. The inclusion of LTPA in model 3 did not change the significances of bonding and bridging social capital predicting self-rated health. Greater LTPA levels were positively associated with self-rated health (*P*-for-linear trends = .007), in which the individuals with LTPA levels of ≥600 MET-min/week showed greater odds of reporting ‘good’ self-rated health when compared to no-LTPA group (OR = 1.97; 95% CI = 1.21–3.21; AME = 0.097). Additional analyses using the KHB method confirmed non-significance of the mediating effects of LTPA on bonding (change in logit coefficients = 0.02; 95% CI = −.13–.17; *P* = .813) and bridging (change in logit coefficients = 0.001; 95% CI = −.15–.15; *P* = .989). Lastly, additional inclusion of SES variables in model 4 showed that both bonding and bridging social capital were no longer significant predictors of self-rated health (*P*’s > .05), while LTPA levels remained the significant in that individuals with ≥600 MET minutes/week had greater odds of reporting ‘good’ self-rated health when compared to no-LTPA group (OR = 1.83; 95% CI = 1.11–3.00; AME = 0.084). There was a significant linear trend by household income levels (*P*-for-linear trends = .020), where those with high household income group showed greater odds of reporting ‘good’ self-rated health compared to those with low household income group (OR = 2.14; 95% CI = 1.13–4.05; AME = 0.109).

**Table 3:** The results of hierarchical logistic regression analyses predicting the likelihood of reporting ‘good’ self-rated health

	Model 1	Model 2	Model 3	Model 4
	OR (95% CI)	OR (95% CI)	OR (95% CI)	OR (95% CI)
Personal social capital				
Bonding social capital	1.17^*^ (1.08–1.26)	1.14^*^ (1.04–1.24)	1.11^*^ (1.02–1.22)	1.09 (0.99–1.19)
Bridging social capital	1.04 (0.98–1.12)	1.09^*^ (1.01–1.18)	1.09^*^ (1.01–1.18)	1.08 (0.99–1.17)
Demographic variables				
Age (ref: < 60 years)		1.96^*^ (1.28–3.04)	2.04^*^ (1.32–3.18)	1.95^*^ (1.26–3.04)
Gender (ref: male)		1.90^*^ (1.18–3.06)	1.99^*^ (1.22–3.23)	2.06^*^ (1.26–3.36)
Race/ethnicity (ref: white)		1.09 (0.63–1.93)	1.03 (0.59–1.85)	1.02 (0.58–1.80)
Marital status (ref: single or no partnership)		1.22 (0.81–1.84)	1.23 (0.81–1.86)	1.04 (0.67–1.62)
Chronic diseases (ref: no)				
Yes, one		0.37^*^ (0.22–0.60)	0.37^*^ (0.22–0.61)	0.38^*^ (0.23–0.64)
Yes, one or more		0.10^*^ (0.06–0.16)	0.10^*^ (0.06–0.17)	0.10^*^ (0.06–0.18)
*P*-for-liner trends		<.001	<.001	<.001
LTPA (ref: no-LTPA)				
< 600 MET-mins/wk			0.98 (0.59–1.63)	0.94 (0.57–1.57)
≥ 600 MET-mins/wk			1.97^*^ (1.21–3.21)	1.83^*^ (1.11–3.00)
*P*-for-liner trend			.007	.017
Household income (ref: low)				
Middle				1.29 (0.79–2.12)
High				2.14^*^ (1.13–4.05)
*P*-for-liner trend				.020
Education (ref: low)				
Middle				0.98 (0.58–1.67)
High				1.33 (0.72–2.45)
*P*-for-liner trend				.361
Nagelkerke *R*^*2*^	.076	.256	.274	.292
Goodness of Fit (*P-*value) ^a^	.230	.290	.100	.414

*OR* Odds ratios, *CI* Confidence intervals, *LTPA* Leisure-time physical activity, *MET* Metabolic equivalent tasks

^a^*P*-value from the Hosmer-Lemeshow goodness-of-fit test

^*^P < .05

The follow-up examination of the model including higher-order interaction terms between social capital and SES variables demonstrated a significant interaction effect between bridging social capital and household income (*P*-for-interaction effect = .012). The results of simple effect logistic regression analyses by household income levels are presented in Table 4 (the results from hierarchical logistic regression analyses for each group are reported in Supplement Tables 1, 2 and 3). Among individuals with low and high household incomes, bridging social capital were positively associated with self-rated health (OR = 1.19; AME = 0.035 and OR = 1.29; AME = 0.017, respectively). Bonding social capital was also significantly associated with greater odds of reporting ‘good’ self-rated health among those with low household income (OR = 1.18; 95% CI = 1.02–1.37; AME = 0.034). Both bonding and bridging social capital were not significant predictor of self-rated health among individuals with middle household income; yet, LTPA was significantly associated with greater odds of reporting ‘good’ self-rated health in that group (*P*-for-linear trends = .033).

**Table 4:** The results of multivariate logistic regression analyses stratified by household income levels

	Low (*n* = 200)	Middle (*n* = 252)	High (*n* = 210)
	OR (95% CI)	OR (95% CI)	OR (95% CI)
Personal social capital			
Bonding social capital	1.18^*^ (1.02–1.37)	1.04 (0.90–1.22)	0.95 (0.76–1.19)
Bridging social capital	1.19^*^ (1.03–1.37)	0.98 (0.86–1.11)	1.29^*^ (1.06–1.56)
Demographic variables			
Age (ref: < 60 years)	1.99 (0.92–4.30)	2.07^*^ (1.00–4.28)	2.69 (0.97–7.44)
Gender (ref: male)	3.64^*^ (1.41–9.39)	1.64 (0.75–3.59)	1.55 (0.55–4.41)
Race/ethnicity (ref: white)	1.37 (0.54–3.47)	0.68 (0.26–1.76)	0.87 (0.20–3.75)
Marital status (ref: single or no partnership)	1.70 (0.78–3.71)	0.88 (0.44–1.79)	0.50 (0.14–1.80)
Chronic diseases (ref: no)			
Yes, one	0.50 (0.21–1.16)	0.37^*^ (0.16–0.86)	0.16^*^ (0.05–0.54)
Yes, one or more	0.08^*^ (0.03–0.23)	0.09^*^ (0.04–0.21)	0.07^*^ (0.02–0.31)
*P*-for-liner trend	<.001	<.001	<.001
LTPA (ref.: no-LTPA)			
< 600 MET-mins/wk	0.81 (0.34–1.94)	0.71 (0.31–1.63)	1.95 (0.56–6.79)
≥ 600 MET-mins/wk	1.02 (0.42–2.49)	2.38^*^ (1.07–5.29)	1.66 (0.58–4.77)
*P*-for-liner trend	.969	.033	.343
Education (ref: low)			
Middle	0.75 (0.34–1.67)	0.92 (0.39–2.21)	1.86 (0.29–11.90)
High	1.90 (0.61–5.95)	1.62 (0.62–4.21)	1.14 (0.20–6.44)
*P*-for-liner trend	.271	.322	.879
Nagelkerke-*R*^*2*^	.375	.276	.254
Goodness of Fit (*P-*value) ^a^	.709	.600	.719

The results of hierarchical logistic regression analysis for each group are reported in Supplement Tables 1, 2 and 3

The model predicted the likelihood of reporting ‘good’ self-rated health

*OR* Odds ratios, *CI* Confidence intervals, *LTPA* Leisure-time physical activity, *MET* Metabolic equivalent tasks

^a^*P*-value from the Hosmer-Lemeshow goodness-of-fit test

^*^P < .05

## Discussion

The present study sought to investigate the links between social capital and self-rated health among middle-aged and older adults while exploring the roles of LTPA and SES on its relationship. In line with previous research, the findings demonstrated a positive relationship between social capital and self-rated health. After adjusting for study confounders including demographic characteristics as well as history of chronic disease, greater bonding and bridging social capital were significantly associated with better self-rated health. This suggests that both stronger connections within homogeneous groups and weaker relations between different groups enhance the feeling of health among middle-aged and older adults, which is aligned with argumentation of Putnam [43] stating that bonding social capital may enhance the experience of health through mobilization of reciprocity and solidarity, and on the other side, bridging social capital may have health effects through external benefits secured.

However, after controlling for SES characteristics in the model, the associations of bonding and bridging social capital with self-rated health were attenuated and lost their significances, indicating that SES may play a crucial role on the relationship between bonding social capital and self-rated health. Whitley [44] explained this phenomenon by considering qualitative approaches, which suggest that social capital can play a minor role in protecting some aspects of health, but this pales into relative insignificance when wider SES factors are considered. Following this argumentation, prior studies have suggested that higher levels of social capital may simply be an epiphenomenon of more influential socioeconomic processes [45]. The follow-up test of interaction effects revealed significant moderating role of household income on the associations between bridging social capital and self-rated health. We found that, among individuals with low household income, bonding social capital was positively associated with greater self-rated health; yet, these associations were not apparent among those in middle and high household incomes. Our findings support one of the hypotheses in the literature, called the “buffer effects”, which suggests greater health benefits of social capital among people from disadvantaged populations when compared to their counterparts from non-disadvantaged populations [46]. Specifically, our results are aligned with a recent systematic review which concluded that bonding social capital may play a buffering role on the effects of low SES on health [23], suggesting that promoting social supports or networks within the people sharing common social identifies such as family members or close-friends would positively affect self-rated health among the people with low SES.

Additionally, our study demonstrated that the association of bridging social capital with health varied by the levels of household income, where beneficial effects were more apparent among the people with low and high household incomes, but not for those with middle household income. Interestingly, among the people with middle household income, social capital, both bonding and bridging, were not significantly associated with self-rated health, but LTPA, which was not a significant predictor of health among low and high household income groups, was positively associated with self-rated health. It is unclear as to what underlying mechanism explains such variations by household income levels. But given that the people with middle class tend to have different norms and values than other social classes [47, 48], personal human capital such as investment to health by engagement of PA [49], rather than social capital, may be a stronger influential factor promoting better self-rated health among middle-aged and older adults who are in the middle class. Collectively, our findings may imply that social networks and relationships established between the people from dissimilar social identifies may play a positive role in promoting the self-rated health of individuals who are at the lower and higher end of the SES spectrum. However, for those who are in the middle class, individualized behavioral factors such as LTPA rather than building social connectivity may be more influential to subjective health.

In line with previous study [50], engagement in LTPA that meets the current recommendations of the WHO, was found to be significantly and positively associated with better self-rated health. The findings also demonstrated positive associations of LTPA levels with both bonding and bridging social capital, supporting the previous results [51]. However, there was insufficient evidence observed to claim the mediating role of LTPA on the relationship between social capital and self-rated health, in which the inclusion of LTPA in the model did not significantly alter the association of bridging social capital with self-rated health even after the analysis was stratified by annual house income level (results are not reported but provided in Additional files). Our findings are generally aligned with former results from Boen et al. [52] who reported insignificant mediating effects of LTPA on the relationship between the indicators of social capital and self-rated health among middle-aged and older adults (≥55 years old). However, the present results are in contrast with the results from Mohnen et al. [53] who found PA as the behavioral factor at individual-level mediating the positive associations of neighborhood social capital and self-rated health among adults ≥18 years old. As previously noted, social capital is a broad concept and its operational definition is largely inconsistent across the studies [14, 34], which makes a direct comparison of the results with previous studies difficult. In other words, the discrepancies of the results may be attributed to the differences in measurement of social capital including the level of focus (e.g., individual- or, neighborhood-level social capital), dimension (e.g., cognitive/structural social capital, or bonding/bridging social capital), and measurement methods (e.g., surrogate indicators or structured questionnaire). In this study, social capital was operationally defined from the network perspective and measured using a structured questionnaire assessing bonding and bridging social capital at individual-level [26], which have been considered as part of structural social capital [14]. However, some researchers suggest that cognitive social capital, which refers to what people feel regarding social relations such as norms of trust and reciprocity, is more strongly associated with self-rated health [11], implying that the role of LTPA on the relationship between social capital and health may likely be influenced by how the social capital is operationally defined and measured.

The present study contributes to the body of literature by identifying socio-ecological factors that enhance self-rated health among middle-aged and older adults, helping to better understand the process of successful aging. However, caution is necessary when interpreting our findings due to several methodological limitations. First, as previously discussed, the interpretation of the results concerning social capital should be limited to bonding and bridging social capital as measured by PSCS. Second, the present study hypothesized LTPA as a potential mediating factor explaining the links between social capital and self-rated health based on the previous literature showing the directional association of social capital with LTPA (i.e., social capital → LTPA). However, it is also highly plausible that PA and social capital are reciprocally associated in that greater LTPA may lead to greater social capital as reported in the previous studies [54, 55]. This implies that LTPA may not be the complete mediator but a confounding factor influencing the association of social capital with health. Additionally, there is a growing body of literature concerning the potential endogeneity and direction of the association between social capital and health [56]. In other words, people with greater health may have a greater chance to engage in social activities that can improve the sense of social capital, which is, however, not aligned with the direction of the association hypothesized in the present study. Social capital is still a growing topic of research in the field of public health [14] and future studies with robust experimental or longitudinal designs would be promising to better understand the mechanisms explaining the link between social capital and health. This study also operationally defined one’s SES based on household income and education level following the suggestion of Grundy and Holt [57]. However, it should also be acknowledged that household income may not directly indicate the deprivation level of older adults and thus, the results of the present study, particularly the moderating effects by household income, should be interpreted bearing this limitation in mind. Due to the cross-sectional nature of the present study with limited sample size, we could not further elucidate the causal associations not only between social capital and LTPA but also between other study variables, which we shall recommend being addressed in the future study. Lastly, the survey sample of this study was recruited from the Qualtrics research panel that may not fully represent the target population. Although the Qualtrics panels are shown to provide a reliable and valid survey data, the generalizability of the present findings should be tested in different, larger representative sample of middle-aged and older adult populations.

## Conclusion

The present study highlights that developing new social connections with people from different social identities as well as strengthening ties within closed social networks would be the promising strategies to improve sense of the health in aging societies. Although there was a lack of empirical evidence supporting the mediating role of LTPA on the relationship between social capital and health, greater LTPA was independently associated with better self-rated health, emphasizing the positive role of LTPA for successful healthy aging. Lastly, there is an interaction effect between SES and social capital, in that social capital differentially affects self-rated health across the levels of household income. This suggests that SES differences should be accounted for when formulating and implementing the strategies to promote health in aging societies.

## Supplementary Information

**Additional file 1: Supplement Figure 1.** Marginal effects on the probability of reporting “Good” self-rated health by bonding and bridging social capital across household income level. The figures present the corresponding results reported in Table 4. **Supplement Table 1.** The Results of Hierarchical Logistic Regression Analyses Predicting the Likelihood of Reporting ‘Good’ Self-rated Health Among Low Household Income Group (*n* = 200). **Supplement Table 2.** The Results of Hierarchical Logistic Regression Analyses Predicting the Likelihood of Reporting ‘Good’ Self-rated Health Among Middle Household Income Group (*n* = 252). **Supplement Table 3.** The Results of Hierarchical Logistic Regression Analyses Predicting the Likelihood of Reporting ‘Good’ Self-rated Health Among High Household Income Group (*n* = 210).

## Data Availability

The datasets used and/or analyzed during the current study are available from the corresponding author on reasonable request.

## Abbreviations

CI: Confidence interval
IPAQ: International Physical Activity Questionnaire
LTPA: Leisure-time physical activity
PA: Physical activity
MET: Metabolic equivalent unit
OR: Odds ratio
PSCS: Personal social capital scale
SES: Socioeconomic status
