# Supplementary material for: Personal social capital and self-rated health among middle-aged and older adults: a cross-sectional study exploring the roles of leisure-time physical activity and socioeconomic status
Source: BMC Public Health. 2021 Jan 6;21:48. doi: 10.1186/s12889-020-10043-6 (PMC7789776; doi:10.1186/s12889-020-10043-6)
Supplement: Supplementary file 1 — Additional file 1: Supplement Figure 1. Marginal effects on the probability of reporting “Good” self-rated health by bonding and bridging social capital across household income level. The figures present the corresponding results reported in Table 4. Supplement Table 1. The Results of Hierarchical Logistic Regression Analyses Predicting the Likelihood of Reporting ‘Good’ Self-rated Health Among Low Household Income Group (n = 200). Supplement Table 2. The Results of Hierarchical Logistic Regression Analyses Predicting the Likelihood of Reporting ‘Good’ Self-rated Health Among Middle Household Income Group (n = 252). Supplement Table 3. The Results of Hierarchical Logistic Regression Analyses Predicting the Likelihood of Reporting ‘Good’ Self-rated Health Among High Household Income Group (n = 210). [file 12889_2020_10043_MOESM1_ESM.pdf]

Supplement Figure 1.

Marginal effects on the probability of reporting “Good” self-rated health by bonding and bridging social capital across household income level. The figures present the corresponding results reported in Table 4.

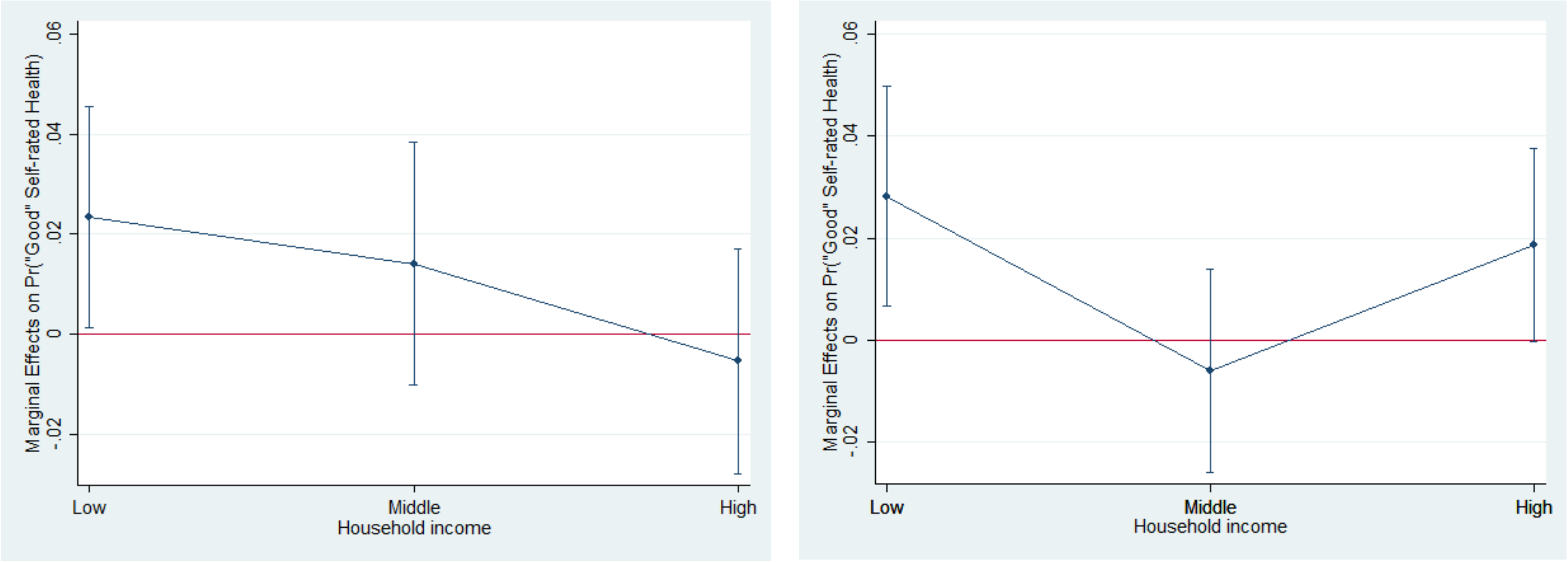

(a) Marginal effects of bonding social capital by household income level

(b) Marginal effects of bridging social capital by household income level

Supplement Table 1.

The Results of Hierarchical Logistic Regression Analyses Predicting the Likelihood of Reporting 'Good' Self-rated Health Among Low Household Income Group (*n* = 200)

	Model 1	Model 2	Model 3	Model 4
	OR (95% CI)	OR (95% CI)	OR (95% CI)	OR (95% CI)
Personal social capital				
Bonding social capital	1.17* (1.08-1.26)	1.18* (1.03 – 1.36)	1.18* (1.02 – 1.36)	1.18* (1.02 – 1.37)
Bridging social capital	1.04 (0.98-1.12)	1.19* (1.03 – 1.36)	1.19* (1.04 – 1.37)	1.19* (1.03 – 1.37)
Demographic variables				
Age (ref: <60 years)		1.92 (0.90 – 4.10)	1.94 (0.91 – 4.17)	1.99 (0.92 – 4.30)
Gender (ref: male)		3.49* (1.38 – 8.84)	3.48* (1.37 – 8.85)	3.64* (1.41 – 9.39)
Race/ethnicity (ref: white)		1.44 (0.58 – 3.53)	1.40 (0.56 – 3.51)	1.37 (0.54 – 3.47)
Marital status (ref: single or no partnership)		1.55 (0.72 – 3.32)	1.55 (0.72 – 3.34)	1.70 (0.78 – 3.71)
Chronic diseases (ref: no)				
Yes, one		0.50 (0.22 – 1.15)	0.52 (0.22 – 1.19)	0.50 (0.21 – 1.16)
Yes, one or more		0.08* (0.03 – 0.22)	0.08* (0.03 – 0.23)	0.08* (0.03 – 0.23)
P-for-liner trends		<.001	<.001	<.001
LTPA (ref.: no-LTPA)				
<600 MET-mins/wk			0.85 (0.36 – 2.00)	0.81 (0.34 – 1.94)
≥600 MET-mins/wk			1.05 (0.43 – 2.56)	1.02 (0.42 – 2.49)
P-for-liner trends			.909	.969
Education (ref: low)				
Middle				0.75 (0.34 – 1.67)
High				1.90 (0.61 – 5.95)
P-for-liner trends				.271
Nagelkerke-R^2	.145	.359	.360	.375
Goodness of Fit (*P*-value) [a]	.230	.507	.712	.709

OR = odds ratios; CI = confidence intervals; LTPA = leisure-time physical activity; MET = metabolic equivalent tasks

[a] *P*-value from the Hosmer-Lemeshow goodness-of-fit test

* *P* <.05

Supplement Table 2.

The Results of Hierarchical Logistic Regression Analyses Predicting the Likelihood of Reporting ‘Good’ Self-rated Health Among Middle Household Income Group (*n* = 252)

	Model 1	Model 2	Model 3	Model 4
	OR (95% CI)	OR (95% CI)	OR (95% CI)	OR (95% CI)
Personal social capital				
Bonding social capital	1.11 (0.98 – 1.27)	1.07 (0.92 – 1.25)	1.05 (0.90 – 1.22)	1.04 (0.90 – 1.22)
Bridging social capital	0.95 (0.85 – 1.06)	0.97 (0.86 – 1.09)	0.99 (0.87 – 1.12)	0.98 (0.86 – 1.11)
Demographic variables				
Age (ref: <60 years)		1.94 (0.97 – 3.88)	2.17* (1.05 – 4.47)	2.07* (1.00 – 4.28)
Gender (ref: male)		1.48 (0.70 – 3.13)	1.69 (0.77 – 3.67)	1.64 (0.75 – 3.59)
Race/ethnicity (ref: white)		0.75 (0.31 – 1.84)	0.75 (0.29 – 1.92)	0.68 (0.26 – 1.76)
Marital status (ref: single or no partnership)		0.85 (0.43 – 1.68)	0.86 (0.43 – 1.72)	0.88 (0.44 – 1.79)
Chronic diseases (ref: no)				
Yes, one		0.40* (0.18 – 0.89)	0.37* (0.16 – 0.85)	0.37* (0.16 – 0.86)
Yes, one or more		0.10* (0.04 – 0.22)	0.09* (0.04 – 0.22)	0.09* (0.04 – 0.21)
P-for-liner trends		<.001	<.001	<.001
LTPA (ref.: no-LTPA)				
<600 MET-mins/wk			0.75 (0.33 – 1.71)	0.71 (0.31 – 1.63)
≥600 MET-mins/wk			2.50* (1.13 – 5.53)	2.38* (1.07 – 5.29)
P-for-liner trends			.024	.033
Education (ref: low)				
Middle				0.92 (0.39 – 2.21)
High				1.62 (0.62 – 4.21)
P-for-liner trends				.322
Nagelkerke-R^2	.015	.223	.265	.276
Goodness of Fit (*P*-value) [a]	.863	.125	.368	.600

OR = odds ratios; CI = confidence intervals; LTPA = leisure-time physical activity; MET = metabolic equivalent tasks

[a] *P*-value from the Hosmer-Lemeshow goodness-of-fit test

* *P* <.05

Supplement Table 3.

The Results of Hierarchical Logistic Regression Analyses Predicting the Likelihood of Reporting ‘Good’ Self-rated Health Among High Household Income Group (*n* = 210)

	Model 1	Model 2	Model 3	Model 4
	OR (95% CI)	OR (95% CI)	OR (95% CI)	OR (95% CI)
Personal social capital				
Bonding social capital	0.98 (0.81 – 1.20)	0.96 (0.78 – 1.20)	0.96 (0.77 – 1.19)	0.95 (0.76 – 1.19)
Bridging social capital	1.22* (1.03 – 1.45)	1.25* (1.04 – 1.50)	1.26* (1.04 – 1.51)	1.29* (1.06 – 1.56)
Demographic variables				
Age (ref: <60 years)		2.70* (1.00 – 7.32)	2.57 (0.94 – 7.02)	2.69 (0.97 – 7.44)
Gender (ref: male)		1.47 (0.53 – 4.06)	1.57 (0.56 – 4.40)	1.55 (0.55 – 4.41)
Race/ethnicity (ref: white)		0.81 (0.2 – 3.22)	0.81 (0.20 – 3.37)	0.87 (0.20 – 3.75)
Marital status (ref: single or no partnership)		0.51 (0.15 – 1.77)	0.52 (0.15 – 1.85)	0.50 (0.14 – 1.80)
Chronic diseases (ref: no)				
Yes, one		0.16* (0.05 – 0.53)	0.17* (0.05 – 0.55)	0.16* (0.05 – 0.54)
Yes, one or more		0.08* (0.02 – 0.30)	0.08* (0.02 – 0.31)	0.07* (0.02 – 0.31)
P-for-liner trends		<.001	<.001	<.001
LTPA (ref.: no-LTPA)				
<600 MET-mins/wk			2.07 (0.61 – 7.06)	1.95 (0.56 – 6.79)
≥600 MET-mins/wk			1.79 (0.64 – 5.05)	1.66 (0.58 – 4.77)
P-for-liner trends			0.269	.343
Education (ref: low)				
Middle				1.86 (0.29 – 11.90)
High				1.14 (0.20 – 6.44)
P-for-liner trends				.879
Nagelkerke-R^2	.068	.232	.246	.254
Goodness of Fit (*P*-value) [a]	.650	.256	.359	.719

OR = odds ratios; CI = confidence intervals; LTPA = leisure-time physical activity; MET = metabolic equivalent tasks

[a] *P*-value from the Hosmer-Lemeshow goodness-of-fit test

* *P* <.05
